# Acute Lymphatic Leukæmias

**Published:** 1905-03-04

**Authors:** 


### ACUTE LYMPHATIC LEUKÆMIAS.

Among the mysterious blood diseases acute lymphatic leukæmia ranks high. It is not a very common ailment, yet in the *British Medical Journal* of February 25th six fresh cases are reported. In none of these cases the age of the patient exceeded 21, and in two cases the disease occurred at the early age of three years. Clinically, the onset of the illness is more or less rapid, its duration varies from three weeks (as in one case of Dr. McCrae's series) to about two months. The diagnosis may be impossible without the aid of a blood examination, but in the majority of cases there is enlargement of lymphatic glands, and a moderate enlargement of the spleen.

The blood-count always shows a deficiency of red cells, and a leucocytosis varying in this series from 26,000 to the enormous figure 783,000 per c.mm. (as in the case reported by Dr. McWeeney). The differential count of leucocytes establishes the identity of the disease by showing that the leucocytic excess is due to hyaline leucocytes. These appear to be most often of the large uninuclear type. Occasionally these cells supply over 90 per cent. of the total leucocytes, and when they do not, the large and small uninuclear cells divide a similar percentage. Polymorphonuclear cells, which bulk so largely in the differential count of health, may almost disappear from the blood, and in no case in the present series rose above 13 per cent., falling not infrequently as low as 3 per cent. It is curious that the colour index is little reduced, a fact indicating that though the number of red cells is diminished, their hæmoglobin-content is relatively increased, a condition constantly associated with pernicious anæmia.

The clinical course of all the cases showed a rapid decline. Spontaneous bleeding from the gums and nose, and varying degrees of subcutaneous hæmorrhage marked them all. In the matter of morbid histology it is the rule to find extensive leucocytic infiltration of all the viscera. Unfortunately, no treatment hitherto suggested appears to influence in any way the rapid progress of the case towards a fatal issue.

